# The Editor's Letter-Box

**Published:** 1915-01-30

**Authors:** T. L. Heath

**Affiliations:** To the Chairman of the Board of Management, Norfolk and Norwich Hospital, Norwich.


Chairman of the Board of Management.

Norfolk and Norwich Hospital, Norwich,

January 2, 1915.

**Letter from the Treasury.**

[Copy.]

Treasury Chambers,

January 11, 1915.

Sir,—In reply to your letter of the 2nd instant addressed to the Prime Minister, I am directed by the Lords Commissioners of His Majesty's Treasury to inform you that they are unable to give any general assurance that relief will be given from public funds in respect of damage incurred through the action of the enemy.—I am, Sir, your obedient servant,

(Signed) T. L. Heath.

